# T-Regulatory Cells and Vaccination “Pay Attention and Do Not Neglect Them”: Lessons from HIV and Cancer Vaccine Trials

**DOI:** 10.3390/vaccines4030030

**Published:** 2016-09-05

**Authors:** Vedran Brezar, Véronique Godot, Liang Cheng, Lishan Su, Yves Lévy, Nabila Seddiki

**Affiliations:** 1 Institut National de la Santé et de la Recherche Médicale (INSERM), U955, Equipe 16, Créteil 94000, France; vedranbrezar@gmail.com (V.B.); eronique.godot@gmail.com (V.G.); yves.levy@aphp.fr (Y.L.); 2 Faculté de médecine, Université Paris Est, Créteil 94000, France; 3 Vaccine Research Institute (VRI), Créteil 94000, France; 4 Department of Microbiology and Immunology, Lineberger Comprehensive Cancer Center, The University of North Carolina at Chapel Hill, Chapel Hill, NC 27599, USA; chengl84@email.unc.edu (L.C.); lishan_su@med.unc.edu (L.S.); 5 AP-HP, Hôpital H. Mondor—A. Chenevier, Service d’immunologie clinique et maladies infectieuses, Créteil 94000, France

**Keywords:** memory cell, Tregs, HIV, vaccine, DC-based vaccine, OX40, CD25, CD39, hu-mice

## Abstract

Efficient vaccines are characterized by the establishment of long-lived memory T cells, including T-helper (effectors and follicular) and T-regulatory cells (Tregs). While the former induces cytotoxic or antibody responses, the latter regulates immune responses by maintaining homeostasis. The role of Tregs in inflammatory conditions is ambiguous and their systematic monitoring in vaccination along with effector T-cells is not instinctive. Recent studies from the cancer field clearly showed that Tregs suppress vaccine-induced immune responses and correlate with poor clinical benefit. In HIV infection, Tregs are needed during acute infection to preserve tissue integrity from an overwhelmed activation, but are not beneficial in chronic infection as they suppress anti-HIV responses. Current assays used to evaluate vaccine-induced specific responses are limited as they do not take into account antigen-specific Tregs. However, new assays, such as the OX40 assay, which allow for the simultaneous detection of a full range of Th-responses including antigen-specific Tregs responses, can overcome these issues. In this review article we will revise the role of Tregs in vaccination and review the recent work performed in the field, including the available tools to monitor them, from novel assays to humanized mouse models.

## 1. Introduction

Vaccination remains the most effective way to prevent and reduce the global burden of infectious diseases [1]. Efficient vaccines are characterized by the establishment of a long-lived memory immunity. 

In order to develop successful vaccines against pathogens such as HIV or HCV, it will be essential for the vaccine to induce not only neutralizing antibodies but also to generate highly effective T cell immunity.

There have been considerable efforts in determining the role of T and B lymphocyte responses in protective immunity [2,3,4]. One goal of therapeutics and prophylactic vaccines is to augment the cytotoxic capacity of antiviral CD4 and CD8 by targeting dendritic cells (DCs) [5,6,7,8,9,10,11,12,13]. These cells have the ability to orchestrate the interplay between innate and adaptive immunity. By targeting the appropriate innate receptors on DCs, it is possible to modulate the functional quality of memory cells. CD4+ memory T cells play an important role in vaccination as they provide crucial help to B and CD8+ T cells [14,15]. They comprise diverse populations, namely Th1, Th2, Th17, T regulatory cells (Tregs), T follicular helper (Tfh), and probably others [16]. 

Tregs are central in maintaining cell homeostasis [17]. Although their role in cancer has clearly been associated with poor clinical benefit [18], their role in HIV infection is ambiguous, as they decrease immune activation, which is beneficial for HIV-infected individuals, but they also suppress anti-HIV responses (reviewed in [19]). Traditional methods used to evaluate antigen-specific responses, including effector cytokine or proliferative capacity measurements are limited, as they do not take into account antigen-specific Tregs, known to be anergic in vitro [20]. Therefore it is important to consider new approaches to define vaccine-induced responses including memory Th1, Th17, Tfh, and Tregs. Moreover, various animal models including humanized mice have been shown to be very useful and provide a robust model for studying human immunity and immunopathogenesis of various pathogens. This review will focus on Tregs in vaccination and will highlight the main work that has been achieved in the field to reveal the role of Tregs in vaccine-induced immune responses and also raise awareness regarding the monitoring of these responses that often fail at detecting their different flavours.

We will particularly address the induction of Tregs in DC-based vaccines as DCs are the conductors of the specific effector and regulatory adaptive responses against pathogens. Targeting these cells represent an important strategy in new vaccine approaches.

## 2. DC-Based Vaccination

DCs occupy a prominent place and attract attention in both prophylactic and therapeutic vaccination, as they are most efficient at capturing, processing, and presenting antigens to T lymphocytes. In preventive vaccines, the help of CD4 T cells is crucial in mounting specific-antibody responses that are able to block the spread of infection [21]. Therapeutic vaccines are designed to elicit powerful cytotoxic T cells required in the elimination of virally infected cells in chronic viral infections or abnormal cells in cancer [22]. 

Immature DCs as sentinels of peripheral tissues control their microenvironment by processing surrounding antigens. The type of immune responses elicited are determined by the presence or the absence of danger signals that come with the antigen. The lack of alert signals results in immune tolerance either through T cell deletion or through the induction of Tregs. With danger signals, DCs become mature, express more MHC-class II and co-stimulatory molecules, and secrete different cytokines that control effector T cell subset differentiation. 

It is commonly accepted that vaccine adjuvants act by inducing the maturation of DCs, as do danger signals [23]. DCs sense vaccines and adjuvants and then program the protective immune responses. Progress in the understanding of DC biology, such as the discovery of functionally distinct DC subsets, benefits vaccine development. Challenges lying ahead are no longer about finding efficient adjuvants to activate DCs but rather to target the right DC subset to elicit appropriate adaptive immune responses. 

### Strategies for DC-Based Vaccination

DC subsets express various patterns of TLRs but also express other pattern recognition receptors (PPRs) such as cell-surface C-type lectins (CLEC9A, DC-SIGN, LOX-1) used to sense pathogens [24]. The targeting of cell-surface endocytic PRRs by chimeric proteins using an anti-DC receptor antibody fused to a selected antigen is one of the principles of DC-based vaccines. The other one is the ex vivo generation of DCs and their injection into the patient after the steps of antigen loading and of maturation. In the former case, the objective is to deliver a small quantity of antigen in vivo directly to the intended DC subset. DC targeting vaccines can potentially be administrated with an adjuvant. What emerges from studies is that the choice of the DC surface target as well as the selection of the adjuvant will have a critical impact on the type of elicited immunity [25]. Ralph Steinman and colleagues initially developed the approach of DC targeting using a monoclonal anti-DEC-205 to deliver antigens to DCs [25]. The conjugation of anti-DEC205 to ovalbumin was the first step to demonstrate the priming of potent CD4+ T cell responses and the cross-priming of CD8+ T cells in naïve mice [26]. They further provided pre-clinical evidence that in vivo DC-targeting together with an adjuvant elicited specific CD4+ T cell responses and a humoral immunity by vaccinating transgenic mice using an anti-human DEC-205 recombinant IgG1 fused to HIV-gag p24, where animal DCs expressed human DEC-205 [27]. Since then, our group described the generation of broad HIV-1 specific CD4+ T responses, the cross-priming of CD8+ T cells, and the induction of humoral immune responses in non-human primates (NHPs) by targeting LOX-1 and DCIR on DCs [7,13]. Immunization of NHP with the humanized anti-human DCIR recombinant IgG4 fused to HIV-1 gag p24 without adjuvant elicits a robust anti-Gag p24 IgG response [7]. Vaccination of NHPs with a humanized anti-human LOX-1 recombinant IgG4 antibody fused to the HIV-1 Env protein (gp140 from clade C 96ZM651) generates multifunctional and broad specific CD4^+^ T cells, a specific CD8+ T cell immunity, as well as specific IgG and IgA responses, in particular when given with poly ICLC as the adjuvant [13]. Human in vitro and preclinical studies in NHPs are promising in the fields of HIV, cancer, and autoimmunity [28]. However, only a few clinical studies testing the immune efficacy of DC targeting vaccines in HIV and cancer are registered on clinicaltrials.gov. The clinical studies reported in the database indicate that DC therapies in HIV infection, cancer, and autoimmune diseases are mostly based on administration of in situ generated DCs. 

In HIV, different DC preparations loaded with different HIV antigens have been tested [29,30]. A recent study used ex vivo generated dendritic cells pulsed with heat-inactivated autologous virus as a therapeutic vaccine in chronic HIV-1-infected patients under ART (anti-retroviral therapy) [8]. Results showed that the DC therapy was safe, well tolerated, and was associated with a partial control of HIV viral replication after ART interruption [8]. We have recently developed a therapeutic DC vaccine based on ex vivo generated interferon-alpha (IFN-α) dendritic cells loaded with HIV-1 lipopeptides [5,31]. The vaccine was administered to chronically infected individuals under ART [31]. Vaccinations were followed by a 24-week treatment interruption period. Results showed a reduced peak of viraemia following ART interruption [31]. Interestingly, the control of viral replication following ART interruption was associated in vaccines with increased polyfunctional effector HIV-specific responses and decreased HIV-specific CD25+CD134+CD39+FoxP3+ Tregs responses [5] (See chapter “Tregs responses in vaccinations” below for more details). 

The foregoing examples show that DC-based vaccination is a growing field that is fed by our understanding of DC biology and also by Tregs and effector cells. Many parameters need to be considered for the design of DC-based vaccines. These include: the biological function of target DCs (induction of humoral and/or cellular immunity or immune tolerance), the selection of antigens and their formulation, and the choice of adjuvants or maturation cocktail for ex vivo generated DCs. Examples of DC-based vaccines used in clinical or experimental studies are listed in Table 1.

## 3. Treg Responses in Vaccination

### 3.1. Assays to Monitor Vaccine Responses

The monitoring of vaccine-induced immune responses has evolved substantially since the early vaccine trials. The goal of all methods applied is to find immune correlates of potential clinical effects and to then be able to either predict or influence vaccine efficacy. The assays routinely used in the different laboratories evolved around three main issues: (a) sensitivity to detect an immune response, (b) comprehensiveness to monitor various aspects of the response, and (c) feasibility linked to cell numbers and to technical issues. It is beyond the scope of this review to describe each assay we use to detect T-cell responses after vaccination as there are many other sources reviewed elsewhere [32]. What is common for these assays is that they rarely consider that Treg responses are also induced by the vaccine. Most commonly, cells from vaccinated subjects are stimulated with peptide pools containing epitopes from the proteins present in the vaccine and/or with viral or cellular lysates. Most of the time, cytokine release (by ELISA/Luminex, Elispot, or ICS) or T-cell proliferation, compared to baseline, is measured and reported as a marker of immunogenicity for any given vaccine. These markers are valuable but they measure limited number of parameters and Treg responses are often omitted. Most of the times bulk Tregs, delineated as CD4+CD25^hi^CD127^low^FoxP3+ cells, are measured by flow cytometry and their effect on Teff responses are measured in suppression assays. However, the antigen-specificity of these Tregs is rarely taken into account. Recently, novel assays measuring activation markers on antigen-stimulated T cells have been developed [20,33,34]. These assays, in the absence of other measurements of Treg activity (such as TGF-b or IL-10 production), could be used as the same activation markers that are often expressed on both Tregs and Teffs after activation. Indeed, we have recently shown that cells stimulated with their cognate antigen express high levels of OX40 molecule along with CD25 [20]. Among these cells, a large proportion is comprised of antigen-specific Tregs, expressing FoxP3+ and CD39+ [19], putative Treg markers. This assay, which is very simple to perform, can be used not just to detect antigen-specific Tregs that are induced by vaccination but also other T helper (Th) responses. Recently, while conducting a pilot study of FLU-vaccine induced responses, we were able to detect not only FLU-specific Tregs but also an important fraction of FLU-specific Tfh cells that was directly correlated with the induction of protective antibodies in vaccinated subjects, unlike “bulk” Tfh cells (manuscript in preparation). These results are reminiscent of our recent findings that antigen-specific Tregs and not “bulk” Tregs correlate with HIV-vaccine induced T-cell responses (manuscript in preparation and [5]). 

These types of more sophisticated assays are of interest especially if used in combination with novel methods such as high-throughput proteomics, high-resolution genomics, and transcriptomics. Understanding the molecular biology underlying vaccines is one of the first steps needed to clarify the complexity of immune responses to vaccines. This approach can be of great use especially when combined with multi-parameter cellular immunology. In recent years, systems biology approaches have provided a novel tool to define immunological correlates. Systems biology uses mathematical modelling to integrate multifaceted datasets and make profiles of immune responses. These data can then be further analyzed and compared with clinical outcomes. This approach might allow for the better design of vaccine candidates, reduction of empiricism, and lessening the costs of failed vaccine candidates. A recent study performed by an international consortium showed that systems biology might allow for accurate modeling of the vaccine responses even before intervention [35]. This is even more relevant in vaccination protocols such as in immunocompromised patients suffering from vaccine-non related disease, or patients under immunosuppressive treatment [36]. 

We have recently embarked on an RNA-sequencing study where HIV-specific CD4+OX40+CD25+ cells will be sorted from chronically HIV-infected patients who had been vaccinated with an HIV DC-based vaccine [5,9]. Outcomes from this study will complete previous analyses and will hopefully reveal novel immunological correlates that have not been determined using classical methodologies.

### 3.2. Tregs and Vaccination against HIV

The role of Tregs in HIV infection is complex. They play a dual role by decreasing immune activation, which is beneficial for HIV-infected individuals, but also by suppressing anti-HIV responses [19]. Even though it is well documented that they play a prominent role in natural infection, the induction of Tregs in HIV-vaccination trials has not been studied extensively. 

Macatangay et al. published a study in which they showed that a dendritic-cell based vaccine loaded with HIV-peptides induced slight increases in Tregs [36]. These Tregs did not correlate with Teff responses but authors showed that their depletion in vitro led to increased polyfunctional CD8+ T-cell responses. Interestingly, this inhibition of the response was shown to be HIV-specific [37]. 

The study of antigen-specific Treg responses is still in its early days. HIV-specific Tregs were just recently reported for the first time by Angin et al. [38]. The authors used MHC Class II tetramer loaded with gag peptide to detect these cells. In clinical trial settings, this approach is challenging mostly due to the genetic variability of MHC Class II as well as the limited availability of Class II tetramers.

To bypass these technical issues, we have recently developed a method by which simultaneous detection of antigen-specific Teffs and Tregs can be achieved after in vitro stimulation with the cognate antigen [5,19,20]. Using this method, we have described for the first time the role of HIV-specific Tregs in an HIV-vaccine trial. The trial involved the use of autologous dendritic-cells pulsed with HIV-lipopeptides. To our surprise, we observed very high levels of HIV-specific Tregs, yielding up to 80% of the specific response in some infected individuals. After vaccination, a shift was observed and HIV-specific Teffs predominated the post-vaccine response. More importantly, this change in the HIV-specific response impacted the magnitude of viral rebound after treatment interruption [5]. In a more recent study, we confirmed our observations and found that HIV-specific, and not bulk Tregs, correlate with clinical outcomes in HIV-vaccination trials (manuscript in preparation). This observation calls for caution, not just in the HIV field but also in cancer immunotherapy trials, where immune modulation is often tempted by agents, that could act not only on desirable Teff responses but also on (tumor-specific) Tregs.

### 3.3. Tregs in Cancer Vaccine Trials

As is the case in HIV infection, the role of Tregs in cancer might be more problematic than expected and probably depends on several factors, such as the type of cancer, stage, etc. [18]. However, several studies performed in mice showed that the depletion of Tregs might be beneficial in cancer settings [39,40,41]. In humans, Chakraborty et al. [42] immunized melanoma patients with MHC class I restricted peptide- or melanoma tumor lysate-loaded APCs and analysed the induced responses. They found significant increases in the number of antigen-specific CD8+ T cells but these responses declined to the pre-vaccine levels by day 28. This loss of CD8+ T cells was associated with the expansion of Treg cells. Even though several tumor antigens are described, tailoring the effective vaccine has been troublesome [43,44]. A potential problem might be the induction of tumor antigen-specific Tregs that might suppress Teff responses as we showed in HIV settings. Indeed, Bonertz et al. [45] studied tumor antigen-specific Tregs in colorectal cancer patients. They found that Tregs’ specific inhibition of Teff responses to certain antigens was much more pronounced than others. This study demonstrated that it could be possible to tailor the vaccine by choosing the antigens to which Treg suppression is less effective (“Tregs-independent antigens”) [45]. Along the vaccination protocols, development of the antibodies against immune checkpoints has recently come into the limelight of cancer research. These two approaches could be complimentary as many targeted molecules such as CTLA-4, CD103, or OX40 are overexpressed on Tregs [46]. This opens the possibility of using the targeted depletion of (antigen-specific) Tregs by antibodies as an adjuvant to the vaccine. Another interesting possibility is targeting the immunosuppressive cytokine IL-35. Recent studies showed that neutralization of IL-35 could lead to Treg cell-restricted deletion of IL-35 production and could limit tumor growth [47]. It can be easily appreciated that the number of Treg-specific agents are increasing with time. This surely testifies the awareness of the research community that Tregs play an important role in vaccine-induced responses and that they should not be neglected. 

## 4. Hu-Mice for Treg Responses Studies

The difficulties in studying vaccine-induced Treg responses result from the markers used in Treg cell identification, the compartments and the stage of human diseases in which Tregs are examined, and the methods of Treg cell quantification [48]. More reliable Treg-specific markers need to be investigated for identifying and purifying the Treg cell subset. In this chapter, we will discuss the advances in humanized mice as they can be used as a good model to study the vaccine-induced (Treg) responses. The reagents and approaches that specifically deplete human Tregs in humanized mice in vivo will be critical to define their functions in human immune responses [49].

### 4.1. Humanized Mouse Models with Functional Human Immune Cells, Lymphoid Organs, and Immunity

Significant progresses have been made in the past 10 years after the generation of several immunodeficient mice that lack NK cells as well as T and B cells. Both BalbC rag-γC-null (DKO) and Nod-Scid (or rag)-γC (NSG or NRG) mice have been reported to efficiently support human immune development from transplanted human hematopoietic stem cells (HSC). Humanized mouse models support effective human T-cell responses to vaccination or to human-tropic virus infection. Remarkably, human CD8 T cells in such humanized mice control EBV infection in human B cells. However, although human B cells seem to be efficiently reconstituted, they appear to have an IgM over IgG bias, probably due to impaired maturation and isotype switching. Several improvements have elevated human IgG responses, including the co-expression of human HLA-A2 and -DR MHC genes in NSG mice and the hydrodynamic expression of human IL4/GM-CSF cytokines in humanized mice [50,51]. Therefore, the impaired human IgG induction in humanized mice can be, at least, partly rescued. 

A major improvement in human immune reconstitution involves the co-transplant of human HSC and autologous human fetal thymus fragments (BLT). The BLT Nod-Scid mice develop higher levels of human T cell reconstitution in all lymphoid organs [52]. HIV-1 infection in NSG-hu BLT mice is immunologically controlled and CTL escape mutants are selected as in HIV-infected humans [53]. In addition, similar levels of HIV-1 neutralizing antibody were detected in infected NSG-hu BLT mice as in HIV patients [54]. However, NSG-hu BLT mice seem to develop graft-versus-host-diseases (GVHD), probably due to transfer of the mature human T cells in human fetal thymus fragments [55]. The model has been improved by depleting human T cells in the thymus fragments prior to transplant. These NSG-hu HSC/TEC mice appear to develop comparable levels of total human blood cells but with no or reduced GVHD progression [56,57]. 

### 4.2. Development and Functional Properties of Tregs in the Humanized Mouse Model

Current humanized mice can mount functional T cell immune responses and are useful for studying the development and function of all human T cells including Tregs. Jiang et al. [58] have reported that functional Tregs are developed in the DKO-hu HSC mouse model. Using multi-color flow cytometry, Tregs were detected in the peripheral blood (PB) at similar frequencies to human or mouse LTs, to be either CD4+CD25+ or CD4+FOXP3+ cells. In the functional suppression assay, CD4+CD25^+/hi^ Tregs are able to suppress proliferation of CD4+CD25− responder T cells upon activation with anti-CD3/28 beads or dendritic cell plus staphylococcal enterotoxin B superantigen. Therefore, Tregs with normal phenotypes are developed in all lymphoid organs of humanized mice, and these Tregs are functional with suppressive activity. Onoe et al. [59] also performed the detailed analysis of Tregs in the NSG-BLT mouse model. They analyzed Treg cell populations in peripheral blood (PB) and T lymphocytes (LT) with CD25, CD127, Foxp3, and Helios expression. Foxp3+Helios+ Treg cells develop normally in human fetal thymic grafts and are present in the PB, spleen, and lymph nodes of NSG-BLT mice. They also reported that CD45 isoform expression was normally reversed in association with thymic egress, post-thymic phenotypic conversion, and suppressive function in the NSG-BLT mice. Billerbeck et al. [60] have generated NSG mice expressing human stem cell factor, granulocyte-macrophage colony-stimulating factor, and IL-3 (NSG-SGM3). NSG-SGM3-hu HSC mice are expected to improve certain hematopoietic lineages such as the myeloid lineage. Although PB CD19+ human B cell frequency is significantly decreased in NSG-SGM3-hu HSC mice, CD45+ human leukocyte, CD3+ human T cell, and CD33+ human myeloid cell frequencies in PB are significantly increased in NSG-SGM3-hu HSC mice compared to NSG-hu HSC mice. Consistent with decreased CD8+ T cell subset, increased CD4+ T cell subset was observed in multiple LTs of NSG-SGM3-hu HSC mice. In addition, CD4^+^ Foxp3+ Treg cell population was significantly increased in PB and LTs of NSG-SGM3-hu HSC mice. These Tregs exhibit similar phenotype and suppressive activity as human PB- or tissue-derived Tregs. In summary, Treg cells develop normally and distribute in all lymphoid organs in currently used humanized mouse models. Their phenotype and suppressive activity is similar to human PB- or tissue-derived Tregs. Therefore, current humanized mouse models are useful for studying Treg development and function in vivo in physiological conditions as well as microbial or viral infections. 

### 4.3. Role of Tregs in HIV-1 Infection in the Humanized Mouse Model

As described above, humanized mice provide a robust model for studying human Treg cell development and function, as well as human immunity and immunopathogenesis of HIV-1 infection. We have developed an HIV-1 infection model [61] using humanized mice [62]. In the pathogenic HIV-R3A acute infection model, HIV-1 viral load reaches its peak around one week post infection and then significantly decreases with substantial CD4+ T cell depletion. High levels of productive infection occurs in the lymphoid tissues. Using this model, we utilized multicolor flow cytometry to analyze Treg cells from HIV-R3A infected DKO-hu HSC mice. We found that Foxp3+ human Tregs were preferentially infected and depleted by apoptosis. We also performed Treg cell depletion from humanized mice to determine the role of Tregs in HIV-1 infection by injecting Denileukin diftitox (ONTAK), which is a fusion protein composed of an IL-2 fragment and toxin [53,63]. When Tregs were depleted before HIV-1 infection by injecting ONTAK, HIV-1 infection was significantly decreased as observed by intracellular p24 staining of T cells and plasma HIV-1 viral load. These results indicate that Tregs are primary target cells for HIV-1 infection and are preferentially depleted by apoptosis induced by HIV-1 infection [19]. In addition, Tregs may contribute to the suppression of anti-HIV immunity and to the efficient HIV-1 acute infection. Interestingly, HIV-1 infection of Treg-depleted humanized mice leads to severe tissue injury and liver fibrosis [63]. Therefore, Treg modulates anti-HIV immunity to protect tissue injury from over-reactive anti-HIV immune responses. HIV-1, on the other hand, exploits this self-protective host mechanism to benefit its own infection during acute infection. 

The role of Tregs in chronic HIV-1 infection is of great importance to understand the immunopathogenesis in HIV-1 infection. Our studies showed that Treg cell depletion from HIV-JRCSF chronically infected humanized mice by ONTAK injection significantly induced enhanced human T cell activation, suggesting that Tregs actively suppressed the immune activation during the chronic phase of HIV-1 infection [64]. In addition, Treg cell depletion resulted in a significant increase in HIV-1 viral infection and replication [63,64], suggesting that immune activation during chronic HIV-1 infection enhanced HIV-1 replication or that the Treg cells directly suppressed HIV-1 replication. 

Tregs are likely to suppress anti-HIV immunity during early infection, contributing to robust viral replication in the acute phase. However, Tregs may also be important in controlling immune activation and HIV-1 replication during chronic HIV-1 infection. Recent reports support this hypothesis that Treg cells directly suppress HIV-1 viral replication [65,66]. Thus, Tregs actively control HIV-1 replication, immune activation, and may slow down disease progression during chronic HIV-1 infection.

## 5. Conclusions

Tregs are central in the maintenance of peripheral tolerance and constitute the most important extrinsic inhibitory mechanism that control T cell responses. It is thus crucial to take them into consideration in vaccine protocol design. Antigen-specific Tregs are likely to be best able at regulating targeted immune responses, therefore their modulation (expansion or depletion) using chemical drugs, antibodies, or cytokines should be part of the future challenges for therapeutic vaccine development. Current available assays used to monitor vaccine-induced immune responses are not optimal as they do not include antigen-specific Treg detection. We have recently shown that a large proportion of Foxp3+CD39+ Tregs are comprised within antigen-specific CD4+CD25+OX40+ cells [19,20] upon a re-encounter with antigen. This assay is very simple to perform and can be used not just to detect antigen-specific Tregs that are induced by vaccination, but also to detect other T helper responses such as Tfh. 

Nevertheless, the different subsets of Tregs, namely thymic derived, tissue resident, and induced peripheral Tregs also have different and important impacts on immune responses. The role of each subset should be determined in order to specifically modulate each of them in a given scenario (autoimmune or viral condition). Recent data from our group has shown that these “global or bulk” Tregs are important in the control of T cell exhaustion [67]. 

As advances in combinatorial immunotherapy have gained tremendous successes in the field of oncology, immunologists and clinicians from the infectious diseases field should encourage the development of prophylactic and therapeutic vaccines.

## Figures and Tables

**Table 1:** Examples of DC-Based vaccines used in clinical or experimental studies.

Therapeutic or Prophylactic Vaccine	DC Vaccines: Ex vivo Generated DCs/Recombinant Antibodies Used to Target DCs	Pre-Clinical, Clinical Orexperimental Studies	Indication	Antigen used in the study to load DCs	Maturation Cocktail Used for ex vivo Generated DCs/Adjuvant used for DC targeting	Effector versus Regulatory Elicited Immune Responses	Refs
	Immature GM-CSF/IL-4 monocyte-derived DCs	Pre-clinical study	Healthy volunteers	Influenza peptide MP and keyhole limpet hemocyanin	none	Antigen-specific inhibition of effector T cell function after injection of immature DCs and appearance of MP-specific interleukin 10-producing cells detected by Elispot.	[6]
Therapeutic	Mature GM-CSF/IL-4 monocyte-derived DCs	Clinical study	Stage IV melanoma	Mage-3A1 tumor peptide	TNF	Significant expansions of Mage-3A1-specific CD8+ cytotoxic T lymphocyte (CTL) precursors assessed in ^51^Cr-release assay.	[12]
Therapeutic	Comparative study of CD34+ HPC-derived Langerhans cells versus monocyte-derived DCs	Clinical study	Melanoma	2 synthetic, HLA-A*0201-restricted, melanoma peptides		LCs synthesize much more IL15 than moDCs and stimulate significantly more antigen-specific lymphocytes with a cytolytic IFN-γ profile detected with tetramers and Elispot assays even without exogenous IL-15.	[11]
Therapeutic	Mature GM-CSF/IL-4 monocyte-derived DCs	Clinical study	HIV-1 infection	Autologous inactivated HIV-1	IL1-β/IL-6/TNF/PGE_2_	A significant increase of IFNγ-secreting T cells is observed by Elispot in response to Gag, Nef, Env gp41 peptides for one kinetic point (week 24 post vaccination) out of the 4 tested (12, 24, 36 and 48 weeks post vaccination).	[8]
Therapeutic	Mature GM-CSF/IFNα monocyte-derived DCs	Clinical study	HIV-1 infection	Lipo-5 (a mixture of five HIV-1-antigen lipopeptides derived from Gag, Pol, Nef)	LPS	The control of viral replication following ART interruption is associated with increased polyfunctional effector HIV-specific responses and decreased HIV-specific CD25+CD134+CD39+FoxP3+ T regulatory responses measured in OX40 assay.	[5,9]
	Anti-human DCIR IgG4 fused to HIV-1 antigen	Pre-clinical study in NHP	HIV-1 infection	Gag p24		In NHP, priming with αDCIR.Gagp24 without adjuvant elicited a strong anti-Gagp24 antibody response after the second immunization.	[7]
	Anti-human LOX-1 IgG4 fused to HIV-1 antigen	Pre-clinical study in NHP	HIV-1 infection	Env gp140	With or without Poly ICLC a TLR3 agonist or GLA a TLR4 agonist	Both CD4+ and CD8+ Env-specific T cell responses were elicited by anti-LOX-1 Env gp140, but in particular the CD4+ T cells detected by intracellular staining and Elispot were multifunctional and directed to multiple epitopes.	[13]
	Anti-human DC-ASGPR IgG4 fused to flu antigen	Experimental study (co-cultures DC:T CD4+ with the vaccine)	Potential application in allergic diseases by replacing flu antigen by an allergen	Hemagglutinin 1 (HA1 subunit of influenza virus A/PR/8/34, H1N1)		HA1 targeting to DCs via DC-ASGPR favors the generation of antigen-specific IL-10-producing suppressive CD4+ T cells that were detected in vitro by ICS.	[10]
